# Chemistry of Antimony in Radiopharmaceutical Development: Unlocking the Theranostic Potential of Sb Isotopes

**DOI:** 10.1002/cplu.202400250

**Published:** 2024-10-08

**Authors:** Aivija Grundmane, Valery Radchenko, Caterina F. Ramogida

**Affiliations:** ^1^ Department of Chemistry Simon Fraser University 8888 University Drive Burnaby, BC V5A 1S6 Canada; ^2^ Life Sciences Division TRIUMF 4004 Wesbrook Mall Vancouver, BC V6T 2A3 Canada; ^3^ Department of Chemistry University of British Columbia 2036 Main Mall Vancouver, BC V6T 1Z1 Canada

**Keywords:** Antimony, Radiopharmaceuticals, Radiochemistry, Isotopes, Radiopharmaceutical therapy

## Abstract

Antimony‐119 (^119^Sb) holds promise for radiopharmaceutical therapy (RPT), emitting short‐range Auger and conversion electrons that can deliver cytotoxic radiation on a cellular level. While it has high promise theoretically, experimental validation is necessary for ^119^Sb *in vivo* applications. Current ^119^Sb production and separation methods face robustness and compatibility challenges in radiopharmaceutical synthesis. Limited progress in chelator development hampers targeted experiments with ^119^Sb. This review compiles literature on the toxicological, biodistribution and redox properties of Sb, along with existing Sb complexes, evaluating their suitability for radiopharmaceuticals. Sb(III) is suggested as the preferred oxidation state for radiopharmaceutical elaboration due to its stability *in vivo* and lack of skeletal uptake. While Sb complexes with both hard and soft donor atoms can be achieved, Sb thiol complexes offer enhanced stability and compatibility with the desired Sb(III) oxidation state. For ^119^Sb to find application in RPT, scientists need to make discoveries and advancements in the areas of isotope production, and radiometal chelation. This review aims to guide future research towards harnessing the therapeutic potential of ^119^Sb in RPT.

## Graphical abstract

Antimony‐119 for radiopharmaceutical therapy: Antimony‐119 (^119^Sb, t_1/2_=38.19 h) is a promising candidate for radiopharmaceutical therapy using Auger electrons. Available literature on ^119^Sb is scarce, with only one successful case of chelation, and no reported in vivo applications. This review compiles relevant information on fundamental chemistry of antimony from the lens of the radiopharmaceutical development process (see picture).

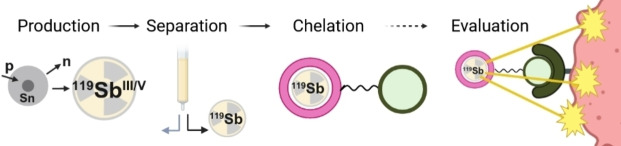

## 1 Introduction

**Figure 1:**
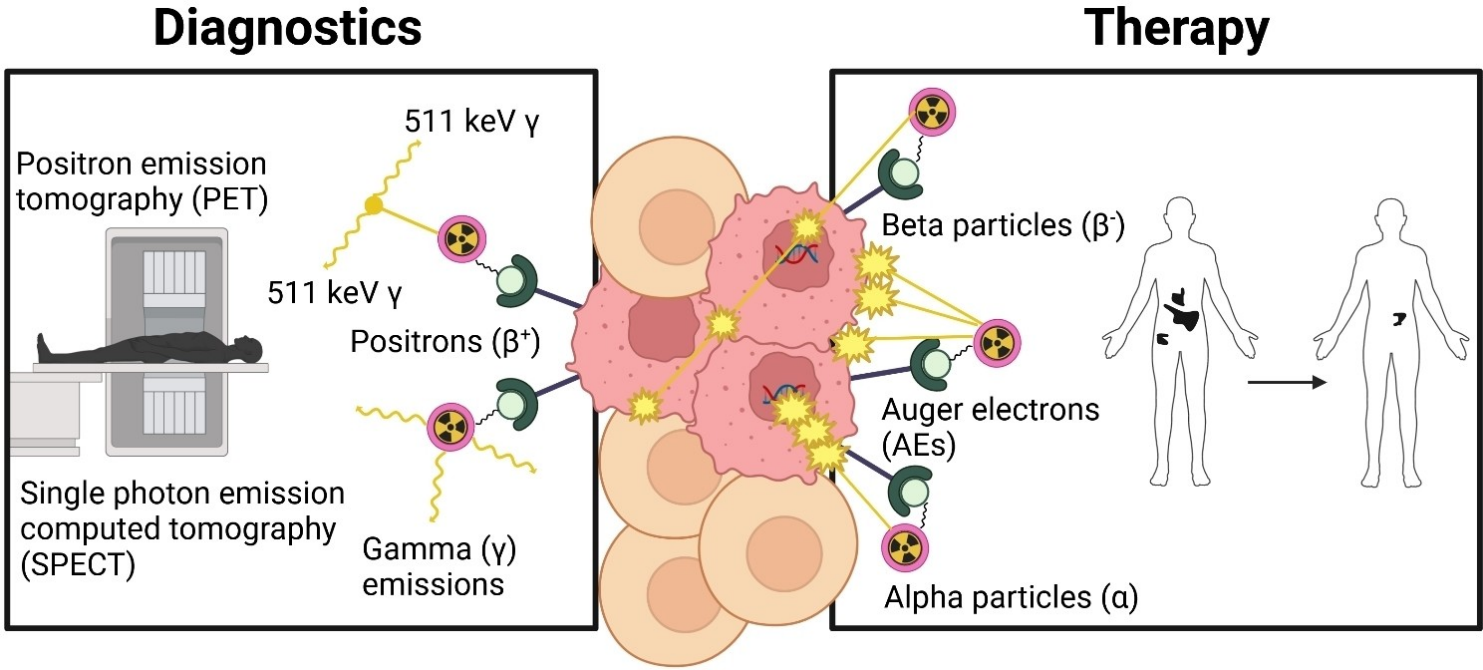
Diagnostic and therapeutic (theranostic) applications of radionuclides in medicine. (Figure created using BioRender.com).

Radiopharmaceutical therapy (RPT) is a rapidly growing field, expanding the pharmaceutical toolbox for cancer imaging and treatment. This technique requires injecting patients with very small amounts of radioactive atoms (aka radionuclides) fused to designer molecules or by themselves. They circulate the body and localize at the desired tissue, where the radionuclide decay emissions are then either captured by imaging detectors surrounding the patient, obtaining diagnostic images, or their energy is deposited close to the site of decay to induce cytotoxic effects for therapeutic purposes (Figure 1).

Historically, the field has predominantly focused on the diagnostic capabilities of the positron (β^+^) and high energy gamma (γ) emitting radionuclides with short half‐lives (minutes to hours), providing essential diagnostic information *via* imaging modalities positron emission tomography (PET) and single photon emission computed tomography (SPECT), respectively. The therapeutic potential of radioactivity has been postulated ever since the discovery of X‐rays, gaining niche use in cancer treatment.[^1^, ^2^, ^3^] Compared to imaging, therapeutic strategies generally involve the use of radionuclides with longer half‐lives (hours to days) and shorter‐range emissions, allowing for deposition of localized dose. There are several suitable types of radioactive decay that can be exploited for this purpose. Beta (β^−^) and alpha (α) particles, as well as Auger electrons (AEs) can be used to inflict damaging effects to cells by inducing DNA damage and generation of reactive oxygen species (ROS), leading to cell death. Amongst other things, the different types of radioactive decay vary in the distance they can travel in tissue and the energy deposited per unit of distance, called linear energy transfer (LET) (Table 1). Radiopharmaceuticals for therapy rely on maximizing this energy deposition; hence they must be delivered as close as possible to the cancer tissue.

**Table 1:** Properties of different types of therapeutically relevant radioactive decay.[^4^, ^5^, ^6^, ^7^]

Type of decay	Linear Energy Transfer (LET) (keV/μm)	Range in tissue (μm)	Range in tissue, measured in 10 μm cells
Beta (β^−^)	0.1–1	2000–10000	200–1000 cells
Alpha (α)	50–230	50–100	5–10 cells
Auger electrons (AEs)	4–26	<0.5	Less than a cell

**Figure 2:**
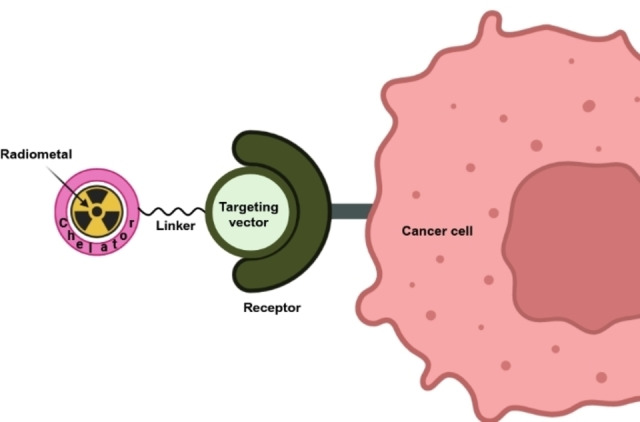
Depiction of the bifunctional chelator (BFC) model for radiopharmaceutical therapy (RPT) using radiometals. (Figure created using BioRender.com).

The relatively long range of β^−^ particles can be exploited by brachytherapy, a more rudimentary approach involving placing a sealed radioactive source near the target tissue. Brachytherapy using β^−^ emitters has garnered much success in prostate cancer treatment.[^8^, ^9^, ^10^] Compared to β^−^ particles, α particles and AEs travel much shorter distances in the body, enabling more selective treatment. The delivery of such radiopharmaceuticals can be challenging, requiring high precision to avoid damaging surrounding healthy tissue. Delivery systems exploiting the properties of certain radionuclides have been utilized clinically, resulting in higher selectivity. For example, iodine‐131 (^131^I, t_1/2_=8.0 d, β^−^=606 keV (I=60 %), γ=364 keV (I=82 %)) naturally accumulates in the thyroid, hence being well suited for treatment of thyroid cancer.[^11^, ^12^, ^13^] Similarly, radium‐223 (^223^Ra, t_1/2_=11.4 d, α=5716 keV (I=51 %), 5606 keV (I=25 %)) dichloride, an FDA approved α radiopharmaceutical relies on bone uptake *via* natural pathways.[^14^, ^15^, ^16^] However, most other radionuclides do not possess these advantageous natural uptake pathways, requiring more sophisticated delivery mechanisms. For radiometals, the most utilized strategy is the bifunctional chelator (BFC) model, where chelator refers to a multidentate ligand used to sequester the radiometal, and bifunctional refers to the ability of a chelator to bind to the radionuclide and be linked to a targeting vector (e. g., small molecule, peptide, antibody), selectively delivering the radionuclide to the tumor (Figure 2). These targeting vectors usually have extremely high affinity (nM range) for proteins overexpressed on the surface of cancer cells, allowing the selective and targeted delivery of the radioactive payload primarily to diseased sites.

### 1.1 RPT with Auger Electrons

Recently, owing to the clinical success of α‐emitting radionuclides such as actinium‐225 (^225^Ac, t_1/2_=9.92 d, α=5830 keV (I=51 %), 5793 keV (I=18 %)), interest in the therapeutic potential of radionuclides with short emission range has grown in rapid strides.^[17]^ Even shorter emission range is achieved by AEs, which can travel a distance of only a fraction of a cell (Table 1). Typically, several AEs are released with each decay of a radionuclide, averaging between 5–30 AEs emitted per decay. While the energy transferred by each individual AE may be lower compared to alpha particles, the higher quantity of particles emitted compensates for this difference, resulting in a substantial dose deposition.[^5^, ^18^] Additionally, their limited range can be advantageous for selective targeting, but it necessitates exceptionally precise delivery mechanisms. The requirement of cell internalization for AE based radiopharmaceuticals is unclear. Direct damage to cell DNA is unlikely to take place when the radiopharmaceutical is bound to the cell surface, as the energy of AEs is most often too low to reach the nucleus if the radiopharmaceutical is not internalized. However, cytotoxic effects may be achieved using AE emitters even without internalization. AEs are often accompanied by conversion electrons (CEs), which generally have a slightly longer range in tissue, equivalent to the cell diameter. The presence of CEs, amongst studies observing the bystander effect (biological response induced in non‐irradiated cells neighboring an irradiated cell) show the promise of AE therapy even if delivery mechanisms involving internalization of the radiopharmaceutical cannot be achieved for the desired radionuclide.[^5^, ^19^, ^20^, ^21^, ^22^]

Several radionuclides have been deemed to be suitable for RPT using AEs (Table 2). There are several desired qualities of an AE emitter for RPT.

**Table 2:** Potential Auger electron (AE) emitters for radiopharmaceutical therapy.

Radionuclide	Half‐life	Average no. of AEs per decay^[a]^			AEs emitted (keV) and their intensity	Accompanying emissions (keV)^[b]^			Ref
		*BrIccEmis* isolated	*BrIccEmis* condensed	MIRD condensed		CEs^[c]^	X‐rays	γ	
^67^Ga	3.26 d	4.56	4.85	4.96	0.99 (168 %) 7.54 (60.7 %)	878	9.57	888	^[23]^
^103^Pd	17.0 d	^[d]^	^[d]^	13.3	2.39 (168 %) 17.0 (18.2 %)	357	23.2	497	^[24]^
^111^In	2.80 d	5.84	7.17	7.43	2.72 (100 %) 19.3 (15.5 %)	245	26.6	245	^[25]^
^119^Sb	38.2 h	10.0	14.4	23.7	2.95 (147 %) 21.0 (11.9 %)	23.9	29.1	23.9	^[26]^
^123^I	13.2 h	7.38	12.3	13.7	3.19 (95.0 %) 22.7 (12.4 %)	625	31.7	1068	^[27]^
^125^I	59.4 d	11.9	20.0	23.0	3.19 (157 %) 22.7 (19.8 %)	35.5	31.7	35.5	^[28]^
^165^Er	10.4 h	^[d]^	^[d]^	7.3	5.33 (65.4 %) 38.4 (4.8 %)	None	55.3	None	^[29]^
^193m^Pt	4.33 d	^[d]^	^[d]^	27.4	7.24 (55.2 %) 51.0 (0.64 %)	135	77.8	136	^[30]^
^197^Hg	2.67 d	^[d]^	^[d]^	23.2	9.70 (92.0 %) 40.3 (3.10 %)	269	80.4	269	^[31]^
^197m^Hg	23.8 h	^[d]^	^[d]^	19.4	7.60 (71.4 %) 53.8 (1.30 %)	161	82.4	164	^[31]^
^201^Tl	3.04 d	10.9	18.8	20.9	7.60 (76.8 %) 53.8 (3.70 %)	167	82.5	167	^[32]^

[a] Calculated using the BrIccEmis code or the MIRD‐RADTABS program, where the isolated atom approach assumes the atom becomes ionized, while the condensed phase approach assumes charge transfer during AE emission, resulting in a neutralized atom^[33]^ [b] Only the highest energy emission is reported [c] CEs – conversion electrons [d] No information found.

1. Large number of AEs emitted per decay: Compared to other types of radioactive decay employed in RPT, AEs have the advantage of emitting many emissions per decay. This should be exploited for use in RPT, hence radionuclides with high number of AEs emitted per decay are desired. Several models have been used to calculate this number and are reported in Table 2. It must be noted that this data varies based on the calculation parameters chosen, and rather than treated as absolute values, the trends should instead be considered.
2. High AE energy: Although they have high LET, by their nature AEs are low energy emissions themselves, generally lower than 25 keV. For maximum therapeutic effect, the energy of AEs for RPT should be on the higher side of the AEs energy range.
3. No accompanying high energy emissions: Most importantly, for use in RPT, absence of any high energy accompanying emissions is required. As mentioned before, low energy CEs can be beneficial, but the presence of any high energy radiation (e. g., gamma rays) will be the limiting factor for selectivity and dosimetry.

To date, there have been no clinical uses of AE emitting radionuclides for RPT. There are, however, certain AE‐emitting radionuclides that have been used in clinical settings because they possess additional decay properties that make them suitable for diagnostic applications. For example, gallium‐67 (^67^Ga, t_1/2_=78.3 h, γ=93 keV (I=39 %), 184 keV (I=21 %), 300 keV (I=17 %)), indium‐111 (^111^In, t_1/2_=2.80 d, γ=173 keV (I=91 %), 247 keV (I=94 %)) and iodine‐123 (^123^I, t_1/2_=13.2 h, γ=159 keV (I=84 %)) all emit AE electrons (Table 2) that can be exploited for therapy, but have primarily been used for scintigraphy and SPECT imaging, given their accompanying photon emissions.[^23^, ^25^, ^27^] While ^67^Ga eventually became obsolete due to the availability of better imaging agents and modalities like ^18^F‐FDG for PET, some niche uses for ^111^In and ^123^I still remain, predominantly for imaging of infections and thyroid scans, respectively.[^34^, ^35^, ^36^, ^37^, ^38^, ^39^] Recently, there has been renewed or heightened interest in these radionuclides because researchers are exploring the therapeutic potential of their emitted AEs.[^40^, ^41^] *In vitro* studies using ^111^In have shown internalization in human neuroblastoma cells and decrease in cell proliferation rate, while preclinical *in vivo* studies have shown tumor growth inhibition and even complete tumor resorption in mice.[^42^, ^43^, ^44^] Similarly ^123^I has shown selective internalization and high nuclear uptake *in vitro*, as well as growth inhibition of pancreatic cancer *in vivo* without notable toxicity to healthy tissue.[^45^, ^46^] As these radionuclides have shown desirable qualities *in vitro/in vivo*, interest in AE emitting radionuclides has grown. The ability to use the same radionuclide for diagnostic imaging and therapy is convenient and simplifies the synthesis of the radiopharmaceutical. However, this means that the limiting factor for dosimetry will be the high energy emissions used for imaging rather than the therapeutic AE emissions, lowering the limit of the therapeutic dose that may be administered. Therefore, radionuclides without an abundance of high energy emissions are preferred for the deposition of a more localized dose, enabling the use of higher doses without the risk of damaging surrounding healthy tissue. Hence, AE emitting radionuclides that have not been utilized before such as mercury‐197 m/g (^197 m/g^Hg, t_1/2_=23.8 h (m), 2.67 d (g)), erbium‐165 (^165^Er, t_1/2_=10.4 h), and antimony‐119 (^119^Sb, t_1/2_=38.2 h) have been brought to the attention of the RPT community.[^26^, ^29^, ^31^, ^47^, ^48^, ^49^, ^50^]

### 1.2 Antimony‐119 and its Theranostic Pairs

**Figure 3:**
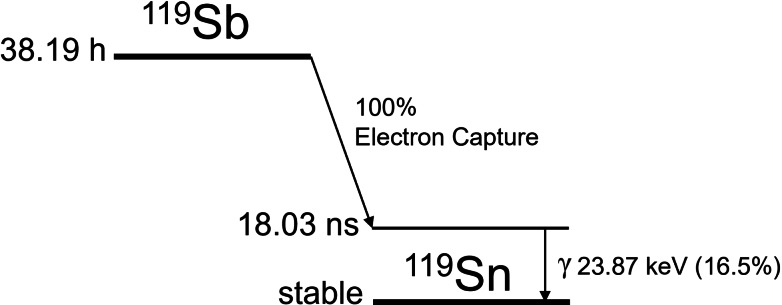
Simplified decay scheme of antimony‐119.

Antimony‐119 (^119^Sb) excels in all aspects desired of an AE emitter for RPT. It decays primarily *via* the emission of AEs and low energy CEs, with negligible contributions of low energy X‐rays and one low energy γ emission (Table 2).^[26]^ It decays directly to stable tin‐119 (^119^Sn) (Figure 3), which is favorable for a radiopharmaceutical as it eliminates the risk of a radioactive daughter nuclide de‐chelating *in vivo* and depositing non‐selective dose.

**Figure 4:**
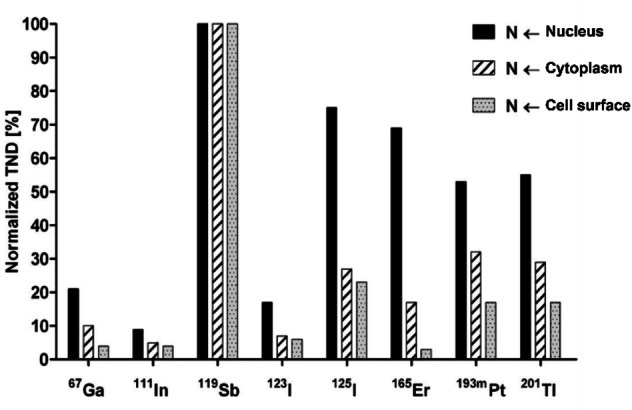
Tumor‐to‐normal‐tissue dose ratio (TND) in different tumor cell compartments relative to normal tissue. Results are normalized for ^119^Sb. Adapted from Ref.^[50]^ Copyright (2008) with permission from Wiley.

As expected by the nature of its emissions (Table 2), the theoretical tumor‐to‐normal‐tissue dose ratio is excellent compared to other AE emitting radionuclides, representing a very localized dose with minimal damage to the surrounding healthy tissue (Figure 4).[^50^, ^51^] Hence, ^119^Sb, absent of any other accompanying emissions, holds the key to achieving cancer treatment on a cellular basis.

Due to the lack of accompanying photon emissions of appropriate energy, the detection of ^119^Sb in the body would not be possible. Therefore, therapeutic radionuclides such as ^119^Sb are often combined with radionuclides suitable for imaging, forming what is called a theranostic radionuclide pair. Having a theranostic radionuclide pair of the same element is especially advantageous, as it ensures that both the diagnostic and therapeutic drugs have matching biodistribution, pharmacokinetics, and pharmacodynamics, ensuring that the response to therapy and dosimetry calculations can be accurately predicted by the imaging companion. So called “chemically‐matched” diagnostic radionuclide candidates for ^119^Sb are antimony‐117 (^117^Sb, t_1/2_=2.80 h, γ=158.45 keV (I=86 %)) and the higher energy meta‐stable state of antimony‐118, antimony‐118m (^118m^Sb, t_1/2_=5.0 h, β^+^=147 keV (I=0.16 %)), having suitable emissions for SPECT and PET, respectively.[^52^, ^53^]

Several studies and reviews have postulated the viability of the ^119^Sb/^117^Sb pair for theranostics; however, the lack of a robust radiochemical purification method for either isotope and subsequent limited literature on chemistry (e. g., chelators) capable of attaching the radioantimony to a drug delivery vector have precluded *in vitro* or *in vivo* evaluation of this theranostic pair.[^47^, ^48^, ^54^, ^55^] Consequently, for the potential of Sb‐based radiopharmaceuticals to be tested experimentally, robust radiochemical purification methods for the radionuclide, along with identification of chelators able to complex radioantimony under suitable conditions amenable for radiopharmaceutical elaboration are desperately needed.

This review aims to further explore the fundamental chemistry of antimony from the lens of radiopharmaceutical development to provide an outlook on the future directions for purification and chelation development, in the hopes of bringing ^119^Sb (and its theranostic pairs) one step closer to experimental therapeutic evaluation *in vivo*. In Section 2, the important considerations for Sb‐based radiopharmaceutical development are laid out, and the primary literature available to date on ^119^Sb production, purification, and chelation are summarized. Section 3 provides an in depth look at the general chemistry of Sb. Speciation plays an important role in the chemistry of antimony, and is discussed in the context of toxicological, biodistribution, redox and hydrolysis properties of Sb, highlighting the necessary precautions that must be undertaken in each step of radiopharmaceutical synthesis. Section 4 reviews the literature on available Sb compounds employed in other areas of chemistry and medicine, summarizing their properties, and assessing how this knowledge can be exploited when designing Sb radiopharmaceuticals. Finally, the Summary and Outlook section poses a recommended approach for Sb radiopharmaceutical design based on all the information discussed.

## 2 Considerations for Antimony‐Based Radiopharmaceutical Development

### 2.1 Cyclotron Production

Radionuclides for medical applications are commonly produced using cyclotrons – proton or deuteron bombardment of target material (i. e. stable (non‐radioactive) starting material) that can undergo suitable nuclear reactions, yielding the desired radionuclide, e. g. ^119^Sb as the end product. For use in RPT, a high radionuclidic purity and suitable quantity on the order of Gigabecquerels (GBq; 1 Bq=1 disintegration [nuclide decay] per second) of ^119^Sb are required. When several production routes are feasible, priority must be given to the route that undergoes nuclear reactions with high cross‐sections at given particle energy (i. e., high probability of the reaction occurring at the energy of incoming particles produced by the cyclotron) to yield high amounts of ^119^Sb radioactivity. This reaction is encouraged by choosing the right target material at high purity, which will undergo the desired nuclear reaction. Generally, lower energy cyclotrons (<30 MeV) are preferred, as the probability of undesired nuclear reactions occurring increases with increased energy, affecting the radionuclidic purity. High purity of target material is beneficial for both production of high amounts of ^119^Sb (more chance for the desired nuclear reaction to take place) and high radionuclidic purity (lower amount of other radionuclides produced from impurities). Generally, targets are prepared in their metallic (solid) state. Importantly, for ^119^Sb to be of radiopharmaceutical value, the concentration of stable Sb (^121^Sb and ^123^Sb, referred to in text as ^nat^Sb) present after production must be lower than that of ^119^Sb, as even nanograms of target material remaining present can lower the molar activity (referring to the ratio between the radioactive compound and its isotopically stable analogue present per mole of radiopharmaceutical, measured as Bq/mol) by a significant amount, resulting in non‐radioactive analogues of the radiopharmaceutical, delivering ^nat^Sb to the tumor, and reducing the efficacy of the radiopharmaceuticals.

**Figure 5:**
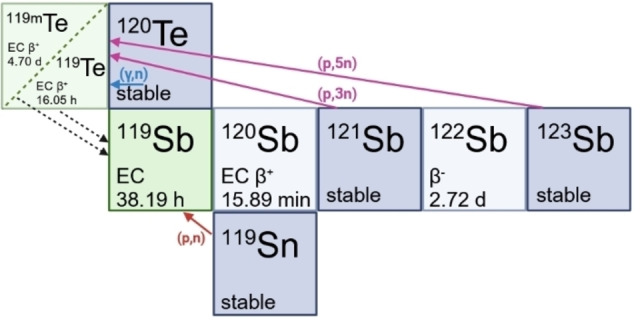
Different possible production routes of ^119^Sb from different target materials (dark blue), showing the direct production route (red arrow), and production of the ^119m^Te/^119^Sb generator *via* cyclotron production (magenta arrow) or the photonuclear reaction (blue arrow). (Figure created using BioRender.com).

Several routes are available for production of ^119^Sb (Figure 5). Proton bombardment of tin‐119 using the ^119^Sn+p→^119^Sb+n reaction (short form denoted as ^119^Sn(p,n)^119^Sb) has a suitable cross‐section (1100 millibarns [mb] at 11–12 MeV proton energy) for direct production of ^119^Sb in low energy medical cyclotrons but requires enriched target material, which can be expensive.[^56^, ^57^, ^58^] Production of a parent isotope that decays to ^119^Sb, called generator production, is also possible, producing tellurium‐119m (^119m^Te, t_1/2_=4.70 d) by proton bombardment of ^nat^Sb, using the ^121^Sb(p,3n)^119m^Te and ^123^Sb(p,5n)^119m^Te reactions.[^26^, ^59^] ^119m^Te decays to ^119^Sb by electron capture (EC), allowing for higher‐scale production and larger supply to hospitals, as the generator can extend the useful lifetime of the radionuclide post production. Although ^nat^Sb is an inexpensive target material (~15 USD per 100 mg target of 99.999 % purity) compared to enriched ^119^Sn (1500 USD per 100 mg target), using ^nat^Sb as the target material can make the subsequent purification steps more difficult as described above. It is important to note that milligrams to grams of target material are used, producing nanograms to micrograms of ^119^Sb (1 GBq of ^119^Sb is equivalent to 39.2 nanograms), which makes the separation on such different scales very difficult and potentially resulting in low molar activity. Production of ^119^Sb using the ^120^Te(γ,n)^119m^Te photonuclear reaction has also been hypothesized, although no experimental work has been done to explore the viability of the route.^[48]^ Hence, production from enriched ^119^Sn targets is generally preferred, despite the higher cost and shorter time of utility.

### 2.2 Separation from Target Material

After production, ^119^Sb, to be used in radiopharmaceuticals, must be separated from the target material. Typically, nanograms to micrograms of the radionuclide are produced on the radiotracer scale, requiring separation from milligrams or even grams of target material. This makes the separation extremely challenging, as the separation efficiency must be very high to remove sufficient amounts of target material (see above). Therefore, radiochemical separation is usually performed in several steps – dissolution, debulking, fine purification and preconditioning.^[60]^ The first step, target dissolution is generally done in concentrated acid to ensure fast, complete dissolution of the metal. For ^119^Sb separations, conc. hydrochloric acid (HCl) is preferred, as both Sn and Sb are readily soluble in it, forming chloride species suitable for further separation chemistry. The general approach for purification is *via* the use of column chromatography, retaining the radionuclide on the column, and eluting the target material. Even if a sufficient separation is achieved, ^119^Sb should be recovered in a matrix compatible with chelation and conjugation to biomolecules. Chelating agents strong enough to compete with the bifunctional chelators (see below) should not be used in the final matrix, and the elute should be concentrated with the radiometal in a suitable oxidation state and species for subsequent radiolabeling of a chelator or bioconjugate. Finally, the matrix must be physiologically compatible (physiological pH, concentrated, no contaminants) so the radiopharmaceutical can be utilized *in vitro*, and *in vivo*. When using enriched target material, target recycling is required to make the production of the radionuclide fiscally feasible. This generally requires quantitative recovery of target in solution compatible with target manufacturing, generally electroplating. More in‐depth analysis of the criteria for radiopharmaceutical separations can be found elsewhere.^[60]^

As mentioned before, the use of ^nat^Sb targets requires quantitative separation between ^nat^Sb and ^119m^Te as to not contaminate the generator with ^nat^Sb, and to ensure levels of Sb are below toxicity limits. A separation removing more than 99.994 % of the ^nat^Sb target from μg amounts of ^119m^Te has been reported.^[59]^ Although this would normally be considered a superb separation efficiency, having removed 49.997 g out of 50 g of the original target material, this means that 3 mg of target material is still left behind. Compared to the 12 μg of ^119m^Te produced by said target, this is a 250‐fold excess of ^nat^Sb, which would result in less than 0.4 % of the final radiopharmaceutical containing ^119^Sb. Typically, this problem is addressed by eluting the generator several times before ^119^Sb of sufficient purity is acquired.

Sn is relatively non‐toxic to humans, so the separation of ^119^Sb from Sn targets does not necessarily need to be quantitative from a toxicological point of view. Radiochemical separation techniques using column chromatography, and liquid‐liquid extraction have been exploited thus far. Some radiopharmaceutically relevant anion exchange column chromatography methods have been reported for the separation of Sb from Sn.[^56^, ^61^, ^62^, ^63^] While the separations differ in their downsides, the common denominator across all is large elution volumes in matrices that are unsuitable for subsequent target recycling, and, most importantly, radiolabeling of a chelator or bioconjugate. Similarly, the available liquid‐liquid extraction methods lack a concentrated end product and often have a sub‐optimal recovery yield.[^64^, ^65^, ^66^] These separations and others have been evaluated in more detail by Randhawa and Olson.^[47]^

### 2.3 Chelation and *In Vitro/In Vivo* Studies

The chelator choice is also critically important when designing any radiometal‐based pharmaceutical. If the chelator can selectively bind the radionuclide in the presence of target material, the separation only needs to be good enough to remove the bulk target below toxicological levels. If the chelator is not specific to the radionuclide, and may bind the target material as well, the separation needs to be immaculate, and the same problem encountered with the ^119m^Te/^119^Sb generator arises. Hence, chelators with high selectivity, tailored to the needs of a particular radionuclide are vital for radiopharmaceutical development.

**Figure 6:**
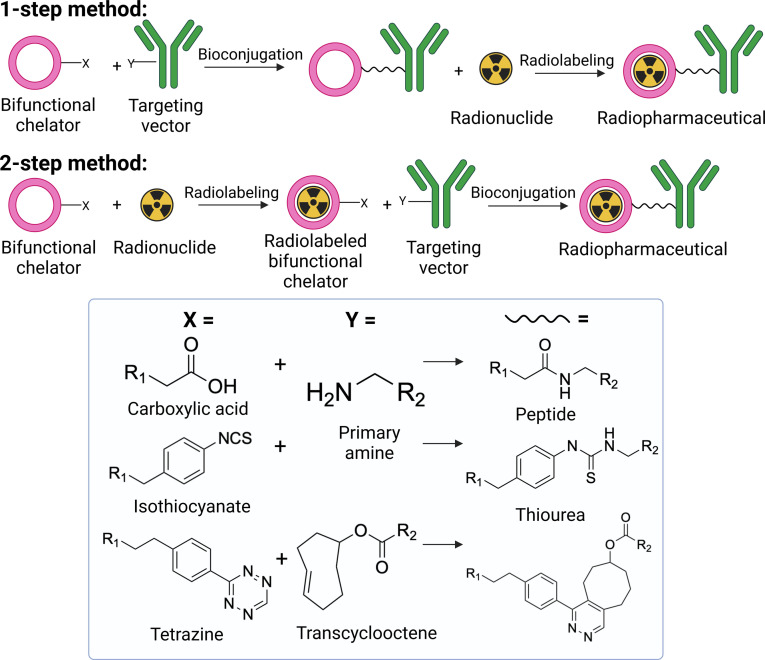
Cartoon depiction of the 1‐step and 2‐step bioconjugation methods for radiopharmaceutical preparation with examples of typical bioconjugation reactions. (Figure created using BioRender.com).

The kinetic inertness of an inorganic ^119^Sb complex *in vivo* must be very high for radiopharmaceutical use due to the many competing endogenous proteins and ligands found in the body, such as L‐glutathione (a small S‐containing tripeptide found in mM concentrations in cells with high binding affinity for certain metal ions such as Sb; see below). Macrocyclic (closed‐chain) chelators are especially preferred as the macrocyclic effect provides extra stability. The chelator must also have a point of functionalization which allows covalent attachment to a biomolecule (targeting vector). This bifunctionalization usually entails the addition of a chemical handle on to the chelator, which can react with available amino acid side chains or other synthetically engineered groups on the biomolecule. Commonly used methods of bioconjugation have been discussed elsewhere.[^67^, ^68^, ^69^] There are two fundamental approaches – bioconjugation of the chelator and targeting vector before adding the radionuclide or after, called the 1‐step and 2‐step methods, respectively (Figure 6). In most cases, the bioconjugate is prepared first, so that the radionuclide is added as the last step, to avoid losses from radioactive decay (1‐step labeling).

**Figure 7:**
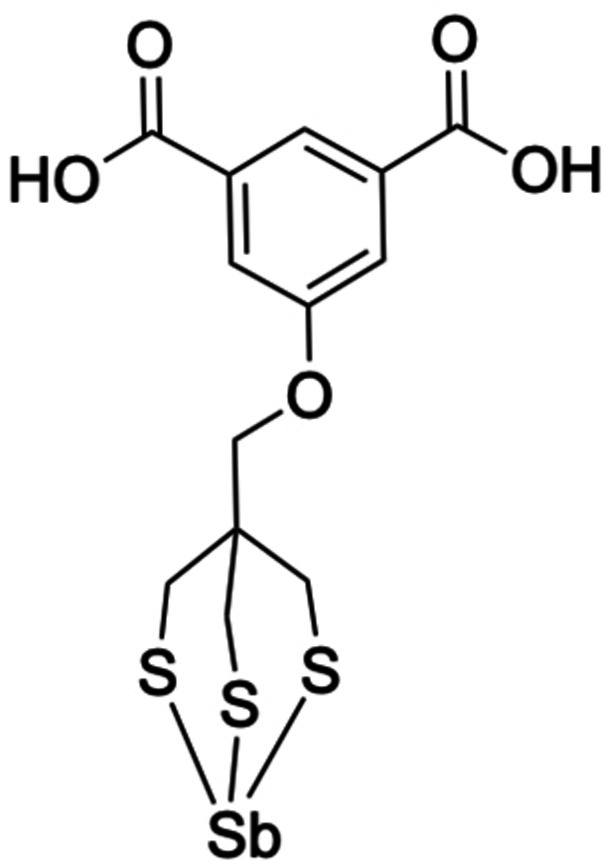
Structure of ^119^Sb labeled 5‐((2,6,7‐Trithia‐1‐stibabicyclo[2.2.2]octan‐4‐yl)methoxy)isothalic acid (^119^Sb(III) trithiol).

To date, only one chelator, 5‐(3‐mercapto‐2,2‐bis(mercaptomethyl)propoxy)isophthalic acid (aka trithiol), has been published that is able to selectively chelate ^119^Sb in the presence of excess Sn, forming 5‐((2,6,7‐trithia‐1‐stibabicyclo[2.2.2]octan‐4‐yl)methoxy)isothalic acid (Figure 7).^[70]^ The solid‐state crystal structure of the ^nat^Sb‐complex shows Sb(III) binding to three thiol (−SH) groups, indicating the 3+ oxidation state and thiol donors on the chelator as desirable characteristics for ^119^Sb pharmaceuticals in the absence of any contradicting data. No macrocyclic or bifunctional chelators, and consequently, no *in vitro* or *in vivo* studies have been reported.

## 3 Chemistry of Antimony

### 3.1 General Properties

Antimony is a pnictogen with two stable isotopes ^121^Sb (57.2 %) ^123^Sb (42.8 %) and is most commonly found in nature in the Sb(III) and Sb(V) oxidation states. Sb has a density of 6.691 g cm^−3^ at 25 °C and a melting point of 631 °C. Relevant properties of Sb are listed in Table 3.

**Table 3:** Properties of antimony.^[71]^

Oxidation state	Coordination number	Radius (Å)^[a]^	Electron configuration
0		1.82 (atomic) 1.41 (covalent) 2.20 (van der Waals)	[Kr] 4d10 5 s2 5p3
3+	3	0.67±0.03	[Kr] 4d10 5 s2
	4	0.75±0.02	
	5	0.81±0.01	
	6	0.86±0.02	
5+	3	0.38±0.01	[Kr] 4d10
	5	0.54±0.01	
	6	0.60±0.01	

[a] Unless otherwise denoted, reports Shannon ionic radii acquired from a database that utilized machine learning to determine ionic radii for different coordination numbers.^[72]^

Being a metalloid, Sb can form a range of different compounds. Sb is generally classified as a borderline Lewis acid based on the Pearson hard soft acid base theory (HSAB), with Sb(V) being relatively harder than Sb(III), meaning Sb(III/V) can have affinity for ligands that are hard Lewis bases, such as carboxylic acids, as well as those that are soft Lewis bases, such as thiols.[^73^, ^74^] Historically, Sb has found use in alloys. One of the first Sb alloys dates to the Bronze age, when small amounts (3–7 wt %) of Sb were added to copper (Cu) to increase the durability and sharpness of Cu tools. At the same time, larger amounts would result in brittle material with an appealing blue coloring for decorative use.^[75]^ We now know to attribute these characteristics to the chemical properties of Sb, namely the bonding between a metalloid and a metal being relatively more ionic and thus affecting the hardness of the alloy, eventually making it more brittle. Currently, the same principle is still applied, and small amounts of Sb are often used in lead (Pb) and Sn alloys to increase the hardness of the alloy, improve the resistance to oxidation and heat due to the higher ionization energy and melting point of Sb. More recently, Sb(III) trioxide (Sb_2_O_3_) has found use in fire retardants in combination with halide compounds. It reacts with the halides under high heat, creating reactive antimony halide compounds that inhibit the fire by reacting with the hydrogen atoms.^[76]^ Antimonides formed with metals like aluminum or gallium are used as semiconductors in electronic devices due to low power consumption ensured by the narrow band gap.^[77]^

### 3.2 Toxicity and Biodistribution

Sb is classified as a priority pollutant by many governing bodies like the European Commission, United States Environmental Protection Agency, and Environment and Climate Change Canada, due to its toxicity and potential carcinogenicity.[^78^, ^79^, ^80^] World Health Organization (WHO) suggests a guideline value of 20 μg/L of Sb in drinking water, and an overall tolerable daily intake of 6 μg/kg of body weight.^[81]^ The total weight average permissible exposure (TWA) and immediately dangerous to life or health (IDLH) values for Sb compounds, regardless of their species have been universally classified as 0.5 mg/m^3^ and 50 mg/m^3^ by most health agencies worldwide. LD50 values for humans were estimated from LD50 values for rats, and generally are in the 101–102 mg/kg range.^[82]^ These toxicological limits are about 5–6 magnitudes higher than the radiotracer amounts typically used for radiopharmaceuticals, which present no toxicological concern. However, the toxicology reveals important information about the biodistribution of Sb and its compounds, and hence will be discussed in this review, highlighting the biological considerations when trying to develop a radiopharmaceutical that is stable *in vivo*.

#### 3.2.1 Environmental Toxicology

**Figure 8:**
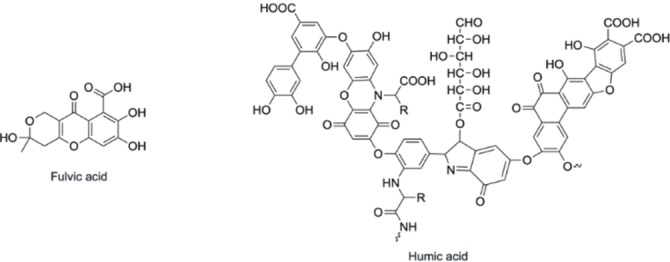
Chemical structures of fulvic and humic acids.

The toxicity of Sb varies based on the oxidation state and species. Elemental Sb is known to be more toxic than its salts, inorganic Sb species more toxic than organic Sb species, and Sb(III) about 10 times more toxic than Sb(V).^[83]^ In plants, uptake of both Sb(III) and Sb(V) has been observed, although the routes of uptake differ. Sb(III) is transported through the cell membranes using water channels, while the mechanism of Sb(V) is unclear, although it has been shown to bind to the hydroxyl functional groups of cell walls.[^84^, ^85^] Sb(III) trichloride (SbCl_3_), Sb_2_O_3_, as well as Sb(V) species found in natural waters (predominantly Sb(OH)_6_^−^) have all been found to be toxic for water fleas.^[86]^ Sb_2_O_3_ was found to be moderately toxic (EC_50_=3.01 mg/L), while SbCl_3_ (EC_50_=423.45 mg/L) and Sb(OH)_6_^−^ (EC_50_=63.8 mg/L) had low toxicity.[^87^, ^88^, ^89^] Although aquatic toxicology of Sb is generally the best investigated, studies on the mechanism of Sb toxicity to aquatic organisms are scarce, and often disagree with each other. Several studies have observed that exposure to Sb resulted in oxidative stress.[^89^, ^90^, ^91^, ^92^, ^93^, ^94^, ^95^, ^96^] One observed that exposure to Sb resulted in an increase in free radicals, which significantly increased the malondialdehyde levels, causing oxidative stress to the bio‐organisms, while another showed that activation of oxidative stress reporter genes was observed in the presence of 11 different Sb compounds.[^89^, ^90^] Such consistent induction of oxidative stress in different aquatic organisms upon exposure to several Sb compounds across different studies suggests oxidative stress may be an underlying indirect mechanism of Sb genotoxicity by causing oxidative DNA damage. Other potential mechanisms, such as DNA inhibition have also been suggested, however, there is disagreement between studies.[^90^, ^91^, ^97^] The toxicity can also be affected by the change in pH and the presence of other compounds, such as dissolved organic matter (fulvic and humic acids; Figure 8) and cations (Ca^2+^, Mg^2+^, or Na^+^). An increase in the pH can inhibit Sb toxicity by competing with the Sb(OH)_6_^−^, while cations and/or dissolved organic matter will reduce the amount of Sb available for direct reaction with any organisms.^[89]^ Sb(III) has been found to selectively bind to humic and fulvic acids in soils (pH 2–10), likely predominantly through their carboxyl and hydroxy‐carboxyl groups, with some binding to other available functional groups, and possibly forming bidentate complexes, which is also likely the case here.^[98]^ This suggests such Sb complexes are stable enough to be inert in the presence of bio‐organisms such as the water flea and hence provides strong evidence for the benefits of using carboxylic and hydrocarboxylic donor atom ligands for radiopharmaceuticals as stability upon delivery is paramount.

#### 3.2.2 Carcinogenicity of Antimony Compounds

Few studies in mammals have been conducted. SbCl_3_ and Sb_2_O_3_ have been found to be genotoxic in human lymphocytes, with the former inducing DNA damage and cell apoptosis.^[99]^ Sb_2_O_3_ and Sb(III) trisulfide (Sb_2_S_3_) have been found to be carcinogenic in rats by inhalation, resulting in lung tumors. These compounds have been classified as possibly carcinogenic to humans and unclassifiable, respectively.[^100^, ^101^] Quantification of the carcinogenic potential of most Sb compounds is currently not possible due to lack of animal studies to date, however caution is generally advised. The mechanism of Sb toxicity in mammals is not known either, although similarly to aqueous organisms, there is some evidence to suggest oxidative stress derived DNA damage.[^99^, ^102^]

#### 3.2.3 Biodistribution

**Figure 9:**
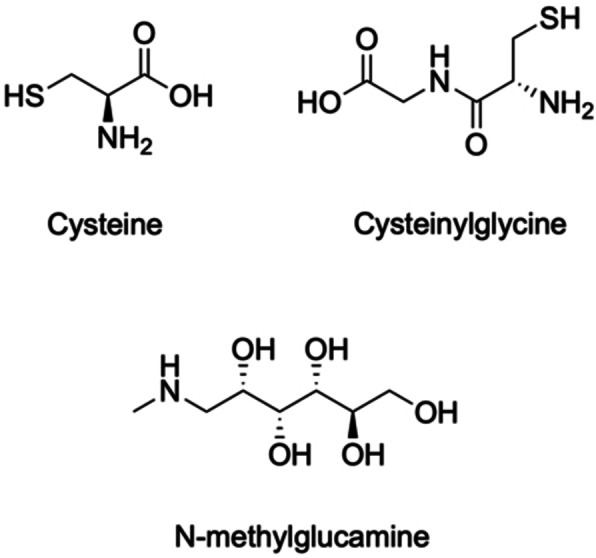
Chemical structures of select compounds that interact with Sb *in vivo*.

In the human body, Sb uptake is generally the highest in the lungs and gastrointestinal tract. The absorption and distribution of Sb in the body is also determined by its oxidation state and speciation. Uptake of Sb(III) is higher in the liver and tends to be excreted in the feces, while Sb(V) is excreted in the urine and has higher skeletal uptake. The main metabolic pathway of Sb has been suggested to be oxidation, eliminating Sb(V) in the urine, while Sb(III) is better retained in the body.^[103]^ In blood, Sb(III) is generally found in red blood cells, and can conjugate to hemoglobin.[^103^, ^104^] The mechanism of conjugation in rat hemoglobin was found to occur at the thiol of the cysteine moiety in the alpha chain of rat hemoglobin.^[104]^ Sb(III) forms a stable complex with glutathione (Table 4), with the thiol group acting as the only binding site, which explains the high liver uptake.^[105]^ Glutathione has also been shown to reduce Sb(V) to Sb(III) in a variety of environments, including humans.[^106^, ^107^, ^108^, ^109^] Reduction of Sb(V) to Sb(III) has also been observed in the presence of other thiol compounds such as cysteine and cysteinylglycine found in the acidic compartments of mammalian cells, trypanothione in Leishmania parasites, and upon administering a mixture of Sb(V) pentachloride and N‐methylglucamine (Figure 9 or Table 5).[^110^, ^111^] Some studies have shown that Sb(III) can enter the cell using membrane proteins like aquaglyceroporins, or hexose transporters.^[112]^

**Table 4:** Select experimentally acquired thermodynamic stability constants for Sb(III) and Sb(V) complexes with hard and soft donor atoms.

Type of complex	Ligand^[a]^	Oxidation state and structure^[b]^	Equilibrium^[c]^	logK^[d]^	Experimental conditions			Ref
					Ionic strength or medium	T (°C)	Type of Measure‐ ment	
Hydroxide	Hydroxide (OH)^−^	Sb(III) 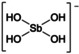	Sb_2_O_3_+3H_2_O⇌2Sb(OH)_3_ Sb_2_O_3_+H_2_O+2H^+^⇌2Sb(OH)_2_^+^ 4Sb_4_O_6_+6H_2_O+4OH^−^⇌4Sb(OH)_4_^−^	−4.44±0.05 −3.3±0.01 −2.21	0 0	20 35	Solubility Solubility	[^188^, ^189^, ^190^, ^191^, ^192^]
		Sb(V) 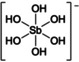	Sb(OH)_5_+H_2_O⇌Sb(OH)_6_^−^+H^+^	−2.5±0.04	0.1 M KNO_3_	25	Glass electrode	^[160]^
Carboxylic acids	Citric acid (H_4_L) 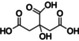	Sb(III) 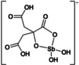	Sb(OH)_3_+H_3_L^−^⇌[Sb(OH)_2_(HL)]^2−^+H_2_O+H^+^ Sb(OH)_3_+H_3_L^−^⇌[Sb(OH)_2_(H_2_L)]^−^+H_2_O Sb(OH)_3_+2H_3_L^−^⇌[Sb(H_2_L)_2_]^−^+2H_2_O+OH^−^	0.1±0.2 4.6±0.3 −3.9±0.3	0	20	Solubility	^[190]^
		Sb(V) 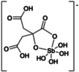	Sb(OH)_6_^−^+H_3_L^−^⇌[Sb(OH)_4_H_2_L]^−^+H_2_O+OH^−^ Sb(OH)_6_^−^+H_2_L^2−^+H^+^⇌[Sb(OH)_4_HL]^2−^+2H_2_O Sb(OH)_6_^−^+HL^3−^+H+[Sb(OH)_4_L]^3−^+2H_2_O	−6.5±0.04 7.8±0.02 8.0±0.04	0.1 M KNO_3_	25	Glass electrode	^[160]^
	Lactic acid (H_2_L) 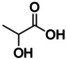	Sb(III) 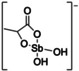	Sb(OH)_3_+HL^−^⇌[Sb(OH)_2_L]^−^+H_2_O Sb(OH)_3_+2HL^−^⇌[SbL_2_]^−^+2H_2_O+OH^−^	1.89±0.03 −5.08±0.04	0	20	Solubility	^[190]^
		Sb(V) 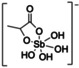	Sb(OH)_6_^−^+HL^−^⇌[Sb(OH)_4_L]^−^+H_2_O+OH^−^	−6.6±0.03	0.1 M KNO_3_	25	Glass electrode	^[160]^
	Oxalic acid (H_2_L) 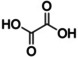	Sb(III) 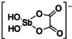	Sb(OH)_3_+HL^−^⇌[Sb(OH)_2_L]^−^+H_2_O Sb(OH)_3_+2HL^−^⇌[SbL_2_]^−^+2H_2_O+OH^−^	3.8±0.2 −5.9±0.1	0	20	Solubility	^[190]^
		Sb(V) 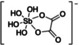	Sb(OH)_6_^−^+HL^−^ ⇌[Sb(OH)_4_L]^−^+H_2_O+OH^−^	−6.2±0.04	0.1 M KNO_3_	25	Glass electrode	^[160]^
	Salicylic acid (H_2_L) 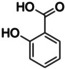	Sb(V) 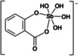	Sb(OH)_6_^−^+HL^−^⇌[Sb(OH)_4_L]^−^+H_2_O+OH^−^	−2.7±0.06	0.1 M KNO_3_	25	Glass electrode	^[160]^
	Tartaric acid (H_2_L) 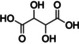	Sb(III) 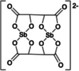	Sb_2_L_2_^2−^+2OH^−^⇌2SbOHL^2−^ Sb_2_L_2_^2−^+2OH^−^⇌2Sb(OH)_3_+2 L_2_^2−^	10.16 6.07	0.1 M NaClO_4′_	20	Glass electrode	^[163]^
		Sb(V) ^[e]^	2Sb(OH)_3_(HL)^−^⇌ Sb_2_(OH)_4_L_2_^2−^	1.26	^[f]^	^[f]^	^[f]^	^[163]^
Amino‐ poly‐ carboxy‐ lic acids	Cyclohexanediaminetetraacetic acid (CDTA) (H_4_L) 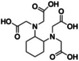	Sb(III) 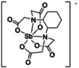	[SbL]^−^+2OH^−^⇌Sb(OH)_3_+HL^3−^	11.24±0.05	0.1 M NaClO_4_	20	Glass electrode	[^163^, ^193^]
	Ethylenediaminetetraacetic acid (EDTA) (H_4_L) 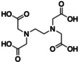	Sb(III) 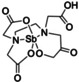	SbO^+^+L^4−^+2H^+^⇌[SbL]^−^ [SbL]^−^+OH^−^⇌ [SbL(OH)]^2−^ [SbL]^−^+H^+^⇌[SbHL] [SbL]^−^+OH^−^⇌[SbL(OH)]^2−^ [SbL(OH)]^2−^+H_2_O⇌[SbL(OH)_2_]^3−^+H^+^ [SbL]^−^+2OH^−^⇌Sb(OH)_3_+HL^3−^	24.8 −8.7 1.02±0.05 8.24±0.05 7.46 12.46±0.05	1 M NaClO_4_ 0.1 M KNO_3_ 0.1 M NaClO_4_	25 25 20	Spectro‐ photometry Glass electrode Glass electrode	[^163^, ^192^, ^194^]
	Hydroxyethylethylenediaminetriacetic acid (HEDTA) (H_3_L) 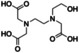	Sb(III)^[e]^	SbO^+^+L^3−^+2H^+^⇌[SbL] [Sb(H_2_L)]^−^+2H^+^⇌SbL [Sb(H_2_L)]^−^+OH^−^⇌SbLOH^2−^ SbL^−^+OH^−^⇌Sb(OH)_3_+H_2_L^2−^ [SbHL]⇌SbL^−^+H^+^ SbL^−^+H_2_O⇌SbLOH^−^+H^+^	20.2 −3.2 −8.1 4.58±0.05 −3.1±0.05 −3.05±0.05	1 M NaClO_4_ 0.1 M NaClO_4_ 0.1 M KNO_3_	25 20 25	Spectro‐ photometry Glass electrode Glass electrode	[^163^, ^192^]
	Pentetic acid (DTPA) (H_5_L) 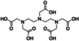	Sb(III) 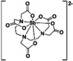	[SbL]^2−^+2OH^−^⇌Sb(OH)_3_+HL^4−^ [SbHL]^−^⇌[SbL]^2−^+H^+^ [SbHL]^−^⇌[SbL]^2−^+H^+^	9.82±0.05 −3.57±0.05 −3.31±0.04	0.1 M NaClO_4_ 0.1 M KNO_3_	20 25	Glass electrode Glass electrode	[^163^, ^195^, ^196^]
	3,6,9,15‐tetraazabicyclo[9.3.1]pentadeca‐1(15),11,13‐triene‐3,6,9‐triacetic acid (PCTA) (H_3_L) 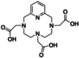	Sb(III) 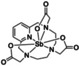	Sb^3+^+L^3−^⇌[SbL] [SbL]+H^+^⇌[SbHL]^+^	23.2 2.40	1 M NaCl	25	Potentio‐ metry, spectro‐ photometry	^[179]^
Thiols	Diethyldithiocarbamic acid (HL_a_) /dithiozone (H_2_L_b_) 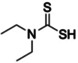 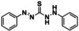	Sb(III) 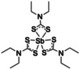	SbL_a_L_b_+2HL_a_⇌Sb(L_a_)_3_+H_2_L_b_	2.47±0.07	CCl_4_	^[f]^	Spectro‐ photometry	[^192^, ^197^]
	NN‐bis(carboxymethyl)dithiocarbamic acid (H_3_L) 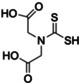	Sb(III) ^[e]^	*Sb+L⇌SbL* *Sb+2 L⇌SbL_2_* *Sb+3 L⇌SbL_3_*	8.91 β_2_ 17.61 β_3_ 5.88	1 M KNO_3_	22	Potentio‐ metry	^[163]^
	dl‐2,3‐dimercaptosuccinic acid (H_4_L) 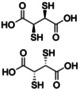	Sb(III) ^[e]^	[SbL]^−^⇌[SbL(OH)]^2−^+H^+^ H_3_SbL_2_^2−^+H^+^⇌H_4_SbL_2_^−^ H_2_SbL_2_^3−^+H^+^⇌H_3_SbL_2_^2−^ HSbL_2_^4−^+H^+^⇌H_2_SbL_2_^3−^ SbL_2_^5−^+H^+^⇌HSbL_2_^4−^ 2[SbL(OH)]^2−^+OH^−^⇌Sb(OH)_3_+SbL_2_^5−^	−4.90 2.57 3.60 4.61 6.82 −10.7	0.1 M NaClO_4_	20	Glass electrode	^[163]^
	meso‐2,3‐dimercaptosuccinic acid (H_4_L) 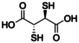	Sb(III) ^[e]^	Sb_2_L_2_^2−^⇌2SbL(OH)^2−^+2H^+^ Sb_2_L_2_^2−^+6OH^−^ ⇌2Sb(OH)_3_+2 L^4−^	13.17 9.2	0.1 M NaClO_4_	20	Glass electrode	^[163]^
	Glutathione (H_3_L) 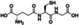	Sb(III) 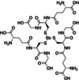	*Sb^3+^+3 L⇌SbL_3_*	25.2±0.4	0.1 M NaNO_3_	25	NMR	^[105]^
	2‐mercaptoethanol (H_2_L) 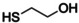	Sb(III) ^[e]^	SbL_2_^−^+3OH^−^⇌Sb(OH)_3_+2 L_2_^2−^	17.98	0.1 M NaClO_4_	20	Glass electrode	^[163]^
	8‐mercaptoquinoline‐5‐sulphonic acid (H_2_L) 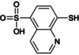	Sb(III) ^[e]^	*Sb+L⇌SbL* *Sb+2 L⇌SbL_2_*	13.7 β_2_ 26.1	^[f]^	^[f]^	Spectro‐ photometry	^[163]^
	Thioglycolic acid (H_2_L) 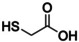	Sb(III) 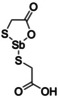	[SbL_2_]^−^⇌[SbL_2_(OH)]^2−^+H^+^ [Sb(HL)_2_]^−^+H^+^⇌[SbL(HL)]	7.58 6.92	0.1 M NaClO_4_	20	Glass electrode	^[163]^
	Thiomalic acid (H_3_L) 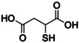	Sb(III) ^[e]^	SbHL_2_^2−^+H^+^⇌SbH_2_L_2_^−^ SbHL_2_^3−^+H^+^⇌SbH_2_L_2_^2−^ SbHL_2_^3−^+3OH^−^⇌Sb(OH)_3_+2 L^3−^	2.4 3.46 5.90	0.1 M NaClO_4_	20	Glass electrode	^[163]^
	Trypanothione (H_4_L) 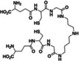	Sb(III) 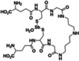	*Sb+L⇌SbL*	23.6±0.4	0.1 M NaNO_3_	25	NMR	[^166^, ^167^]
Phenols	1,2‐dihydroxybenzene (catechol) (H_2_L) 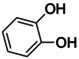	Sb(III) 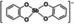	[SbL_2_]^−^+H^+^⇌[SbL(OH)]+H_2_L [SbL_2_]^−^+OH^−^⇌Sb(OH)_3_+2HL^−^	2.37 −5.44±0.01	0.1 M NaClO_4_	20	Glass electrode	^[163]^
		Sb(V) 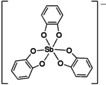	Sb(OH)_6_^−^+3H_2_L⇌[SbL_3_]^−^+6H_2_O	5.5±0.05	1 M KNO_3_	25	Glass electrode	^[160]^
	2,3‐dihydroxybenzoic acid (H_3_L) 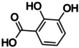	Sb(III) ^[e]^	[SbL_2_]^3−^+H^+^⇌[SbL(OH)]^−^+H_2_L^−^ [SbL]+2OH^−^⇌Sb(OH)_2_+HL^2−^	2.8 −4.17	0.1 M NaClO_4_	20	Glass electrode	^[163]^
	4,5‐dihydroxybenzene‐1,3‐disulphonic acid (tiron) (H_4_L) 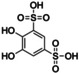	Sb(III) ^[e]^	[SbL_2_]^5−^+H^+^⇌[SbL(OH)]^2−^+H_2_L^2−^ [SbL_2_]^5−^+OH^−^ ⇌Sb(OH)_3_+2HL^3−^ HSbL_2_^4−^⇌SbL_2_^5−^+H^+^ SbL_2_^−^+H_2_L^2−^ ⇌SbL_2_^5−^+2H^+^ *SbL+L⇌SbL_2_* SbL^−^+H_2_L^2−^⇌ SbL_2_^5−^+2H^+^	1.23 −3.95 −2.00 −5.73±0.01 14.5 −6.01±0.06	0.1 M NaClO_4_ 0.1 M KNO_3_ 1 M KCl	20 25 25	Glass electrode Glass electrode Glass electrode	^[163]^
Polyols	Xylitol (H_5_L) 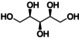	Sb(V) 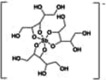	Sb(OH)_6_^−^+3H_5_L ⇌[Sb(H_3_L)_3_]^−^+6H_2_O	5.6±0.05	0.1 M KNO_3_	25	Glass electrode	^[160]^
	Mannitol (H_5_L) 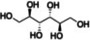	Sb(V) ^[d]^	Sb(OH)_6_^−^+3H_5_L ⇌[Sb(H_3_L)_3_]^−^+6H_2_O	5.6±0.05	0.1 M KNO_3_	25	Glass electrode	^[160]^

[a] Shorthand notation (H_n_L) denotes the fully protonated pro‐ligand with the number of ionizable protons (H_n_) from the ligand (L) [b] Only known structures with either published crystallographic data, or those otherwise deduced in literature are reported here [c] Ligands abbreviated as L in the equations, with the exception of equations involving two different ligands, in which case the ligands are differentiated by the subscript L_a_ and L_b_. Where the formation of the species in equilibrium was not known, simplified equations without accounting for charge balance are listed and are denoted by the use of *italics* [d] Stepwise constants reported unless otherwise denoted as β_x_, which refers to a cumulative/gross constant [e] No known structure found. [f] Not reported.

**Table 5:** Select chromatographic speciation methods for Sb(III) and Sb(V) determination.

Technique	Resin	Functional group	Sample matrix	Dominant species in solution^[a]^	Oxidation state retained	Ref
Anion exchange	Dowex 1X4	Quaternary amine	1.5–2 M HCl	SbCl_4_^−^ Sb(OH)_x_Cl_6‐x_^−^	Sb(III)	^[132]^
	DSC‐SAX	Quaternary amine	Dilute HCl	^[b]^	Sb(III)+Sb(V)	^[133]^
	DSC‐NH2	Aminopropyl	Dilute HCl	^[b]^	Sb(III)+Sb(V)	^[133]^
	Grace Pure SPE Anion‐X	Tetramethyl ammonium	Water	Sb(OH)_3_ Sb(OH)_6_^−^	Sb(V)	^[134]^
Cation exchange	Dowex 50WX8	Sulphonic acid	0.5 M HNO_3_	Sb(OH)_2_^+^ Sb(OH)_5_	Sb(III)	^[135]^
Non‐ionic extraction	Silica	–	Dilute HCl	^[b]^	Sb(III)	^[133]^
	Polystyrene oleic acid imidazole polymer	–	0.1 % w/v diethyl carbamate solution	Sb(III) diethyldithiocarbamate	Sb(III)	^[136]^
HPLC (Anion exchange)	PRP−X100	Trimethyl ammonium	Water	Sb(OH)_3_ Sb(OH)_6_^−^	Sb(III)+Sb(V)	[^127^, ^137^, ^138^, ^139^]
			0–0.1 M citric acid	Sb(III) citrate Sb(V) citrate	Sb(III)+Sb(V)	[^126^, ^139^, ^140^]

**Figure 10:**
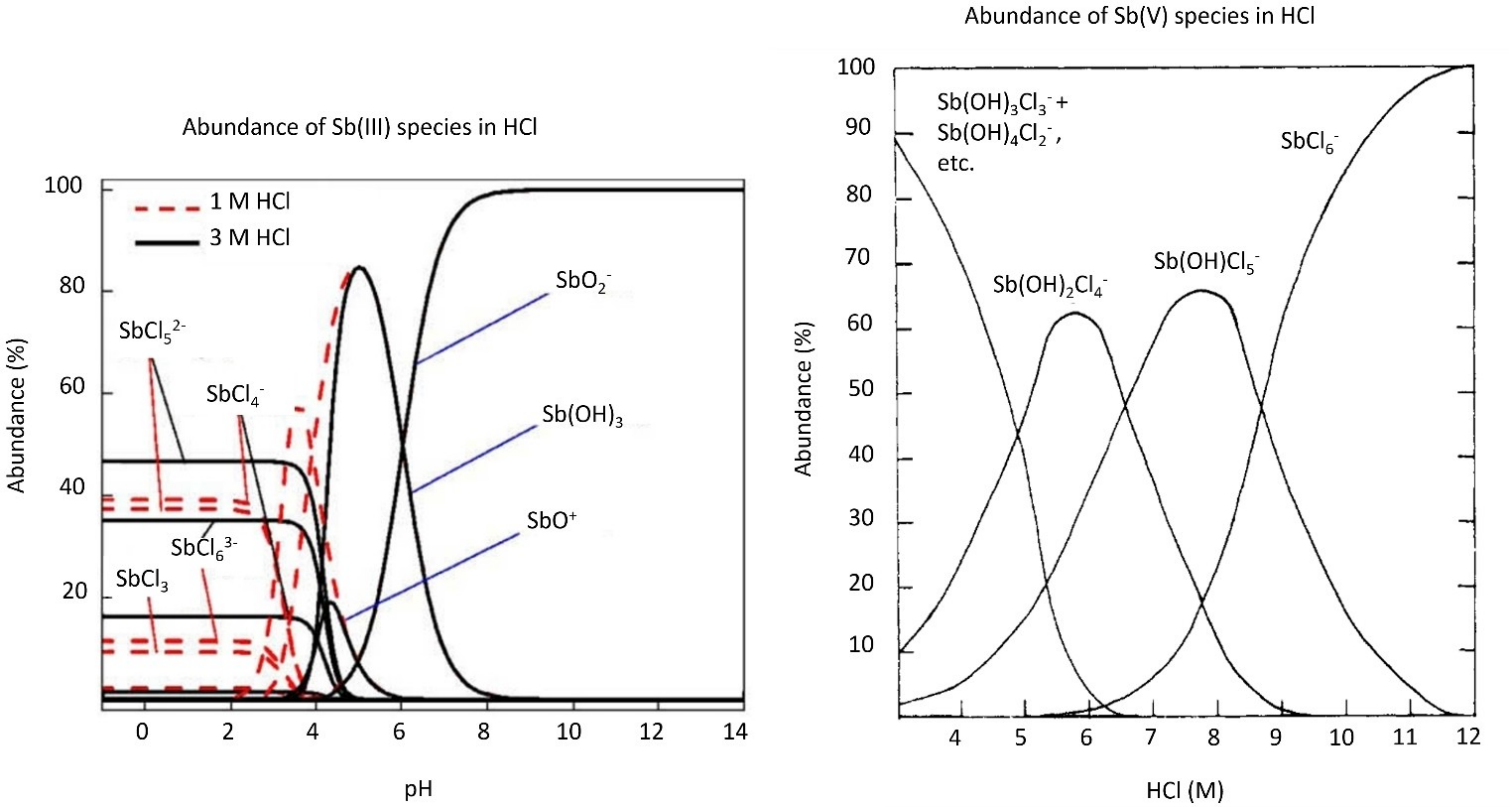
Speciation of Sb(III) and Sb(V) in hydrochloric acid. Adapted from Ref.[^120^, ^121^] Copyright (1954, 2016) with permission from American Chemical Society and Elsevier.

[a] Species elucidated by the authors, or deduced from speciation diagrams (Figure 10), where no suggestions were made [b] Dominant species unclear.

### 3.3 Speciation

Biodistribution, sensitivity to hydrolysis, purification chemistry, and chelator choice are all important considerations in radiopharmaceutical synthesis that depend on the species of Sb. There are many factors that affect the oxidation state and species of Sb. One of the most challenging aspects to overcome in Sb radiopharmaceutical development is hydrolysis. Sb is very sensitive to hydrolysis, a property that has been exploited in hydrometallurgy, achieving near quantitative extraction by hydrolysis at pH as low as 0.5.[^71^, ^72^] Undesired redox reactions can also pose problems, especially when Sb(III) is desired. Very slow oxidation at pH>11 (half‐life in the order of days) of Sb(III) to Sb(V) has been observed in the presence of oxygen (O_2_).^[115]^ Exposure to UV light has been shown to induce oxidation of Sb(III) through increased production of hydrogen peroxide (H_2_O_2_).^[116]^ Oxidation can also be induced by the ionizing radiation emitted by the radionuclide itself, creating reactive oxygen species (ROS), and producing H_2_O_2_.[^117^, ^118^]

Hydrolysis of Sb is a big challenge in radiopharmaceutical development, as the pH and water contents must be carefully considered at each step to avoid the formation of undesired species. Radiation induced oxidation *via* H_2_O_2_ is of concern, emphasizing the speed necessary for radiopharmaceutical synthesis. While O_2_ induced oxidation over time should not pose any problems for radiochemical development compared to the effects of H_2_O_2_, caution is advised when leaving the samples exposed to the atmosphere due to a lack of available kinetic data at low pH. Interestingly, the radiation induced creation of ROS can be a beneficial effect in RPT, further dealing oxidative cell damage to the tumor cells in addition to direct DNA damage.

#### 3.3.1 Speciation Control in Hydrochloric Acid Media

As discussed in Section 2, the Sn targets are dissolved in conc. HCl after irradiation, meaning the dominant species in the initial solution will be Sb with chloride anions. After dissolution Sb is expected to predominantly be in the Sb(III) oxidation state as SbCl_4_^−^ and SbCl_5_^2−^, so for separations requiring Sb(V) an oxidizing agent such as H_2_O_2_ can be added to form SbCl_6_^−^.[^119^, ^120^, ^121^, ^122^] SbCl_6_^−^ readily hydrolyzes, forming Sb(OH)_x_Cl_6‐x_^−^ species at HCl molarity as high as 8 M (Figure 10).^[121]^ The hydrolysis appears to occur more extensively under lower temperatures. The addition of SbCl_3_ has been shown to catalyze the hydrolysis of SbCl_6_^−^, and the exchange between Sb(III) and Sb(V) in the SbCl_6_^−^ form has been noted to occur spontaneously, albeit at a lower rate with increasing acidity.[^123^, ^124^] Theoretical studies have speculated that SbCl_4_^−^, SbCl_5_^2−^, and SbCl_6_^3−^ are the species undergoing hydrolysis below pH 3.5, forming Sb oxychlorides ‐ predominantly Sb_4_O_5_Cl_2_ (Figure 10).^[120]^ There is some evidence to show that Sb(V) is likely extracted as the H_3_OSbCl_6_ adduct species in ether extractions from HCl.[^64^, ^121^] Hence to avoid the inhibition of such separations by reduction and hydrolysis of SbCl_6_^−^, it is paramount to utilize the highest acidity HCl available and conduct the purification process promptly after target dissolution and oxidation.

#### 3.3.2 Speciation Control using Chelating and/or Reducing Agents

The ease of hydrolysis especially complicates the chelation, as typical buffers such as sodium acetate result in the precipitation of insoluble Sb hydroxide species.^[125]^ Hence, the eluent/aqueous phase in any purification scheme must be carefully considered beyond the recovery efficiency. Weak chelating agents such as citrate solutions in water have been used to recover Sb while mitigating hydrolysis by complexing it (Table 4).[^64^, ^126^] In the presence of ammonia buffers, Sb seems to prefer the formation of ammonia adducts over hydrolysis, making them a viable option for chelation.^[125]^ As discussed earlier, Sb(III) may be the preferred oxidation state for a radiopharmaceutical. The addition of EDTA, lactic acid, and citric acid (Table 4) have all been shown to inhibit the oxidation of Sb(III), suggesting them as viable buffers for chelation.[^127^, ^128^, ^129^] If the separation method involves Sb(V), reduction is required before chelation. Some possible reducing agents for Sb are sodium thioglycolate or cysteine (Table 4 or Figure 9). Such complexes could be reasonably transchelated by a multi‐dentate chelator with a stronger affinity for Sb.

#### 3.3.3 Oxidation State Determination

With Sb radiopharmaceutical development currently in its infancy, understanding of the baseline chemistry underlying each step is vital to discriminate between unsuccessful attempts caused by having undesired speciation or unsuccessful attempts caused by unsuitable methodology. Therefore, it is important to know the species of Sb at any given time throughout radiopharmaceutical development. When considering liquid‐liquid extraction, species of Sb can be inferred from whether the element gets extracted into ether or not – Sb(V) is known to be extracted into ethers, while Sb(III) prefers to remain in acidic aqueous phase.[^64^, ^130^, ^131^] Several chromatography methods have been reported to distinguish between Sb(III) and Sb(V) (Table 5).

From this data, it can be concluded that Sb(V) retention occurs primarily when utilizing anion exchange columns, while Sb(III) is able to interact with a wider array of compounds. None of the chromatographic speciation methods listed have investigated Sb speciation in conc. HCl, and therefore have limited use for the speciation of the target solution and an unknown potential for use in the purification step.

Differences in Sb(III) and Sb(V) (0.002 M Sb) in concentrated HCl have been observed spectrophotometrically – Sb(III) solutions are colorless and absorb in the near UV region, Sb(V) solutions are pale yellow and absorb in the visible region, while mixed Sb(III/V) solutions are deep‐orange brown.^[103]^ This enables qualitative speciation determination in concentrated HCl (i. e., prior to purification), although on a scale much higher (g/L) than encountered in radionuclide production (ng/mL to μg/mL) rendering in situ speciation using spectrophotometry unfeasible.

Other more exotic speciation methods like extraction of Sb(III) by magnetizing graphene oxide have also been developed.^[142]^ Another method, square‐wave anodic stripping voltammetry has been utilized for Sb speciation, using up to 3 M HCl as the supporting electrolyte and reported detection limits of 4.21 and 5.05 nmol/L of Sb(III) and Sb(V), respectively.^[116]^ These are potentially workable conditions for speciation determination before and after purification, where the matrix will contain HCl. The authors reported that the Sb(V) signal was noted to suffer and require high concentrations upon hydrolysis and formation of Sb(OH)_6_^−^, encouraging the use of highly acidic media, which should not be a problem for oxidation state determination prior to the purification step. Furthermore, the detection limits reported here should be sufficient if μg amounts of Sb are produced. Sadly, the stripping peaks of Sb(V) and Sb(III) were reported as hard to distinguish, and solutions containing only one or the other oxidation state were recommended for measurement. This heavily limits the use of the technique for radiopharmaceutical development, where a mixture of oxidation states may often be present.

Ultimately, most oxidation state determination methods discussed here require a change in matrix and/or pH, introducing a risk of hydrolysis and oxidation, rendering the measurements not representative of the actual speciation in situ. This highlights the need for more method development, especially within the context of RPT. Still, some of the methods discussed here could be promising as a starting point for developing applicable speciation methods. For example, Sb speciation by HPLC using the PRP−X100 anion exchange column after purification to determine the oxidation state prior to chelation might be utilized if 0.05–0.1 M citric acid at pH<2 is used as the eluent or buffer.

## 4 Antimony Compounds

### 4.1 Antimony Compounds in Medicine

**Figure 11:**
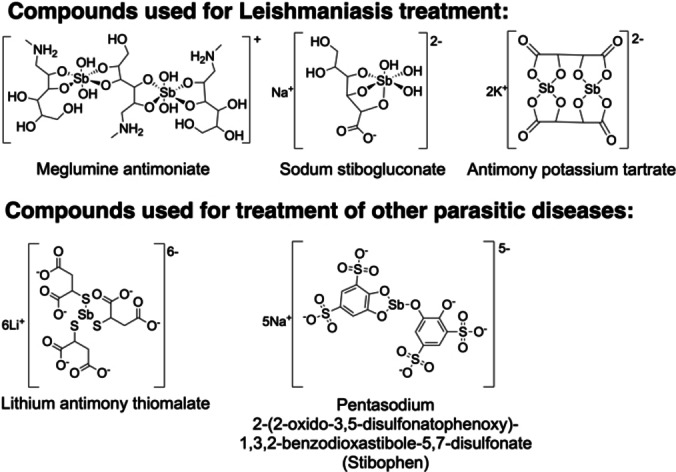
Antimony compounds utilized in medicine for the treatment of several parasitic diseases, predominantly leishmaniasis.

There are several Sb compounds that have been utilized in medicine, predominantly for the treatment of the parasitic disease leishmaniasis. Many Sb(V) compounds, such as meglumine antimoniate and sodium stibogluconate have been used to treat leishmaniasis (Figure 11).[^143^, ^144^, ^145^, ^146^] Neither the mechanism of the drug entry, nor that of the drug action is well understood, but is thought that Sb(V) is reduced to Sb(III) *in vivo*, with thiol compounds participating in the reduction process, particularly trypanothione found in leishmania parasites (Table 4). There is some evidence to suggest that Sb(III) is likely the reactive oxidation state, interfering with the thiol metabolism in the parasite, causing oxidative stress, and eventually cell death, a similar conclusion to that observed in aquatic organisms discussed in section 3.2.[^99^, ^144^, ^147^] Antimony potassium tartrate and lithium antimony thiomalate (Figure 11) have also been used to treat parasitic diseases.[^148^, ^149^]

Anti‐tumour properties of some Sb compounds have been investigated, predominantly those utilized for leishmaniasis treatment, mentioned above. Sodium stibogluconate has been determined to possess some anti‐tumour properties.^[99]^ Antimony tartrate has shown cytotoxic effects in vitro for lung cancer cell lines with similar efficacy as the clinically utilized cis‐platin.[^99^, ^150^] Some triphenylantimony(V) polyamines have also shown cytotoxicity *in vitro* for several cell lines including both HeLa carcinoma cells, and non‐cancer cells.^[150]^

Although the number of Sb compounds in medicine is limited, they encompass a range of different oxidation states and varying donor atom ligands. Stable complexes with O‐donor ligands for pharmaceutical use are achievable in both Sb(III) and Sb(V) oxidation states, although, as evidenced, it is to be expected that the oxidation state *in vivo* will inevitably be Sb(III). Moreover, there is evidence showing the viability of Sb(III) thiolate compounds *in vivo*, widening the range of options available. Sb(III) thiolate compounds are particularly attractive, as such compounds are more likely to be stable enough to prevent transchelation of Sb to endogenous thiolate compounds unlike the O‐donor ligand containing drugs for leishmaniasis treatment.

### 4.2 Organometallic Antimony Compounds

Organometallics of Sb are varied, forming different compounds based on the oxidation state. SbCl_3_ is a starting material for the synthesis of many organometallics and may also be used to form adducts with carbenes.^[151]^ Structurally, radiopharmaceutically relevant organometallic Sb compounds can be designed. Ultimately, organometallic compounds may not be suitable for radiopharmaceutical development due to their considerable synthesis time, during which most of the radioantimony would have decayed, leaving little behind for therapy. Therefore, the inorganic bifunctional chelator model is likely the most desirable way to incorporate Sb into a radiopharmaceutical, where the BFC/bioconjugate synthesis occurs prior to radionuclide production, and the radiometal is added in the very last step prior to radiopharmaceutical injection.

### 4.3 Inorganic Antimony Complexes

#### 4.3.1 Stability of Inorganic Antimony Complexes

Few thermodynamic stability constants for Sb complexes have been reported, and many have been recognized as unreliable by IUPAC.[^152^, ^153^, ^154^] An extensive critical appraisal of low molecular mass organic ligands has been done by Filella and May, reporting thermodynamic data using computer speciation models.^[155]^ Select experimentally acquired stability constants for relevant Sb complexes are listed in Table 4, giving the value acquired at a temperature closest to physiological conditions (37 °C), where several were available.

The ‘borderline’ behaviour of Sb as proposed by the HSAB theory is evidenced by the stability constants listed here, as both stable complexes of O‐donors, and those of S‐donors have been reported. Therefore, both hard and soft Lewis bases will be discussed here.

#### 4.3.2 Inorganic Antimony Complexes with Acyclic Ligands

##### 4.3.2.1 Antimony (Aminopoly−)Carboxylate Compounds

Sb forms stable complexes with many carboxylic and aminopolycarboxylic acids (Table 4). Based on their stability constants EDTA, HEDTA, CDTA, DTPA, lactic, oxalic, tartaric, and citric acids are all viable stabilizing agents for Sb(III). As discussed earlier, some of these acids have already been experimentally proven to mitigate hydrolysis and redox reactions. Furthermore, the use of certain carboxylic acids that naturally occur in the body (e. g. lactic acid) eliminates the need for thorough matrix conversion prior to the administration of a drug, as long as their concentration remains low, or has been appropriately diluted by the end of the radiopharmaceutical synthesis. Therefore, the use of such chelating agents should be prioritized where possible. In fact, the use of a chelating agent as the mobile phase in chromatography is often required, as Sb has been observed to be irreversibly retained on the stationary phase unless a chelating agent is utilized.[^156^, ^157^, ^158^, ^159^]

In theory, weaker interactions between the carboxylic acid and Sb would be preferred to ensure ease of transchelation to a radiopharmaceutically relevant ligand, making lactic acid the most appealing. However, weaker chelating agents can be ineffective in eluting Sb from anion exchange columns, requiring compounds like EDTA and phthalates for elution to occur.^[157]^ Furthermore, weaker interactions often mean that higher concentrations of the chelating agent are required for sufficient stripping of Sb off the column, necessitating further purification steps to remove excess chelating agent prior to radiopharmaceutical administration. One of the most documented carboxylic acid chelating agents for Sb, citric acid, indiscriminately binds to both Sb(V) and Sb(III), which may also be a hindrance when a particular oxidation state is desired.

##### 4.3.2.2 Antimony Polyols

Sb(III) and Sb(V) have been reported to form stable complexes with polyols, forming bonds with the hydroxyl groups in a 4‐ or 6‐coordinate species, respectively.[^160^, ^161^] Such polyol membranes have been used in Sb recovery from wastewater. These membranes could be of interest in Sb purification, as there is no evidence of Sn polyol complexes. However, due to the chelating properties of polyol membranes, the recovery process would likely be challenging, requiring multidentate chelating agents able to form more stable Sb complexes.

##### 4.3.2.3 Antimony Phenols

Another example of the affinity for hydroxyl group donors are phenols. Both Sb(III) and Sb(V) can also form compounds with catechol and its derivatives, forming both inorganic and organometallic compounds.[^160^, ^162^, ^163^, ^164^] In the human body, catechols can be found in human plasma, and are generally bidentate.^[165]^ Therefore, as long as multi‐dentate catechol ligands, presumably with higher stability are used, the naturally occurring catechols should not compete with the radiopharmaceutical in chelating Sb. If suitable catechol chelators are developed, competition studies must be conducted to assess this experimentally.

##### 4.3.2.4 Antimony Thiolate Compounds

Thiol compounds generally act as reducing agents for Sb, reducing any Sb(V) to the Sb(III) oxidation state upon complexation, forming a range of stable Sb(III) thiolate compounds (Table 4). Since Sb(III) is the desired oxidation state, thiol‐based reducing agents, such as thioglycolic acid, may be considered, simultaneously chelating Sb and reducing it, ensuring stable species and uniform oxidation state. In such cases caution must be taken to minimize the concentration of such reducing agents to avoid any toxicological effects, which may be higher than those for carboxylic acids.

The preference for hard versus soft donor atoms becomes apparent when considering molecules that contain both soft and hard donor atoms. In Sb(III) glutathione and trypanothione, Sb binds to the thiol groups rather than carboxylic ones, while in Sb(III) thioglycolate Sb binds to two thiol and one carboxylic group, corresponding to a higher stability of Sb−S bonds (Table 4).[^105^, ^166^, ^167^] Preference for thiols has also been observed in natural organic matter, only undergoing complexation to carboxylic or phenol groups if the thiol content in the soil was low.^[168]^ Furthermore, the only reported stable ^119^Sb labeled complex is ^119^Sb(III) trithiol (Figure 7), providing the only experimental evidence of complexation on a radiotracer scale. For these reasons, chelators with thiol binding sides should be the current focus of ^119^Sb radiopharmaceutical development.

#### 4.3.3 Inorganic Antimony Complexes with Macrocyclic Ligands

**Figure 12:**
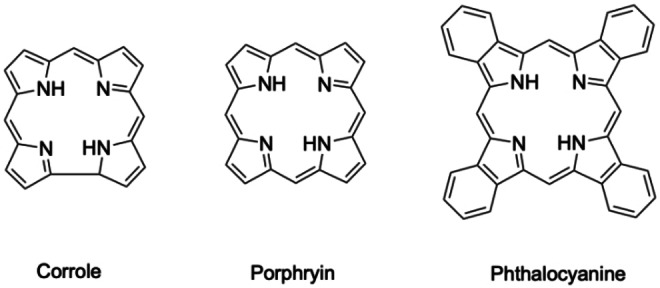
Chemical structures of corrole, porphyrin, and phthalocyanine (Pc) rings.

Antimony forms compounds with various N‐donor macrocyclic ligands. Sb(III) and Sb(V) corroles (Figure 12) have been reported.[^169^, ^170^] Various Sb(III) and Sb(V) porphyrins (Figure 12) have also been synthesized, predominantly as Sb(V) for use as photocatalysts.[^171^, ^172^, ^173^, ^174^, ^175^, ^176^] Numerous Sb(III) and Sb(V) phthalocyanines (Pcs) (Figure 12) have been isolated and characterized, and their premise as photocatalysts shown, although no stability constants of such complexes have been reported. Sb Pcs would likely be very challenging to integrate into Sb radiopharmaceuticals due to the inherent insolubility of metal Pc complexes. Use of porphyrin based metal organic frameworks has been suggested for treatment of wastewater, observing good adsorption of Sb at the porphyrin moieties.^[177]^ While this could prove a promising separation method, similar concerns as for the polyol membranes persist – there is no clear way of recovering Sb in a radiopharmaceutically relevant form after separation.[^178^, ^179^, ^180^] It is also important to note that in Pcs and other N‐donor macrocycles Sb(III) generally sits above the macrocyclic plane, while the Sb(V) equivalents are planar. Such non‐planar complexes tend to undergo π‐π stacking, resulting in polymers of varying length, which is undesirable for a radiopharmaceutical. Therefore, Sb(V) complexes would be preferred, as they would form monomeric radiopharmaceuticals. Unfortunately, Sb Pcs, porphyrins and corroles have the same fundamental problem as Sb organometallics – substantial synthesis time that requires Sb as part of a templated synthesis for the Pc ligand. Synthesis using free base Pc and antimony fluoride is possible, but still requires significant time, harsh conditions and work‐up after synthesis.^[178]^

A macrocyclic aminopolycarboxylate Sb(III) complex, [Sb(PCTA)] has also been isolated (Table 4), although the inertness of the complex was found to be low in near physiological pH, making it unsuitable for *in vivo* applications.^[179]^ To date it is the only macrocyclic Sb(III) complex that is able to chelate Sb after the complete synthesis of the chelator (i. e. without being a templating agent for overall synthesis).

### 4.4 Bifunctionalization and Bioconjugation

Since no bifunctional chelators for Sb have been reported, there is no direct experimental evidence on what route of bioconjugation is best. However, some information can be inferred from existing literature. Generally, the 1‐step radiolabeling protocols (Figure 6) are preferred, in the interest of decreasing the radiopharmaceutical development time (and hence maximizing the amount of radioactivity at end of preparation). However, some of the common linker/bioconjugation strategies employed may be unsuitable for ^119^Sb radiopharmaceuticals as the reactive handle exhibits propensity for Sb binding, potentially interfering with radiolabeling. Sb(III) is known to form strong Sb−S bonds, both as Sb(III) and Sb(V), hence making isothiocyanate and its resulting thiourea bond after bioconjugation (Figure 6) an unsuitable bioconjugation strategy for ^119^Sb radiopharmaceuticals. Sb readily forms complexes with thiourea, therefore the binding may not be selective to the chelator, which is important to maintain the stability of the radiopharmaceutical *in vivo*.[^180^, ^181^] Additionally, biomolecules containing thiol groups such as cysteine may result in non‐specific Sb binding to these sites, instead of the chelator. Hence, bioconjugation after chelation of ^119^Sb may be preferred, *via* 2‐step labeling protocols (Figure 6).^[182]^ A potential compatible bioconjugation route for ^119^Sb radiopharmaceuticals may be the inverse electron demand Diels‐Alder (IEDDA) “click” reaction between a tetrazine and transcyclooctene (Figure 6), although experimental evidence is necessary to validate this hypothesis.[^183^, ^184^, ^185^, ^186^, ^187^]

## 5 Summary and Outlook

^119^Sb has shown great theoretical potential for targeted radionuclide therapy on a cellular basis. Suitable production routes of ^119^Sb have been established for both direct and generator production, with the direct production from ^119^Sn being preferred. However, further development in all other steps of radiopharmaceutical production is needed to obtain experimental verification of the therapeutic effects of ^119^Sb. Due to a lack of relevant literature on ^119^Sb, this review has surveyed the available literature on chemistry of ^nat^Sb in other fields, with a focus on applicability in radiopharmaceutical development.

Since the higher toxicity of Sb(III) is not a concern on the radiotracer scale, both Sb(III) and Sb(V) are viable oxidation states for a radiopharmaceutical from a toxicological point of view. When considering biodistribution, Sb(III) is slightly preferred, due to the bone uptake of Sb(V). What cements the preference for Sb(III) in radiopharmaceutical design are the many reported instances of *in vivo* reduction of Sb(V) to Sb(III) by thiol containing compounds causing concern for de‐chelation *in vivo* if the oxidation state is changed and the chelator is unable to outcompete the endogenous thiol compounds and/or re‐chelate Sb in the Sb(III) oxidation state rendering the radiopharmaceutical unstable, and therefore unfit for the clinic.

Methodology available for ^119^Sb separation from Sn targets is limited, and especially lacks compatibility with subsequent radiopharmaceutical development steps. Control of species and oxidation state is paramount, as Sb is known to readily undergo redox reactions and hydrolyze at pH>0.5, making chelation unlikely to occur. Hence, chelating agents are generally required to recover Sb in a desired oxidation state and species. Use of naturally occurring weak carboxylic acid chelating agents at physiological pH (e. g., lactic acid) as eluents can prevent hydrolysis and overcome the compatibility issues for in vivo use, although they generally form stable complexes with both Sb(III) and Sb(V), resulting in a mixture of oxidation states. Since Sb(III) is desired, thiol‐based reducing agents like sodium thioglycolate may be utilized as eluents in low concentrations, ensuring uniform speciation.

Chelation of ^119^Sb is especially underexplored, making it hard to draw solid conclusions on what would constitute an ideal Sb‐chelator. Based on the only successful chelation of ^119^Sb being a thiol‐based chelator, the high stability of ^nat^Sb thiol compounds reported, and the high affinity of ^nat^Sb for thiol compounds *in vivo*, we propose that thiol‐based chelators must be prioritized in radiopharmaceutical development. However, potentially suitable chelators with hard donor atoms, or a combination of hard and soft donor atoms must not be discounted, as there is evidence of such stable complexes.

There are many hurdles yet to be overcome for ^119^Sb radiopharmaceuticals. First, ^119^Sb radiopharmaceutical development requires the establishment of a robust separation method to ensure reproducible chelation results in media suitable for use *in vivo*. This makes the evaluation of chelators possible, enabling tailored development of bifunctional chelators. Only then can *in vivo* studies be undertaken, bringing ^119^Sb one step closer to use in RPT.

## Conflict of Interests

The authors declare no conflict of interest.

## Biographical Information

*Aivija Grundmane obtained her MChem Chemistry degree from University of Surrey (UK) in 2021. She is currently pursuing a Ph.D. under the joint supervision of Dr. Caterina Ramogida and Dr. Valery Radchenko, dividing her time between doing research at Simon Fraser University and TRIUMF (Canada). Her research is focused on the early stages of antimony‐119 radiopharmaceutical development, namely targetry and separation chemistry*.

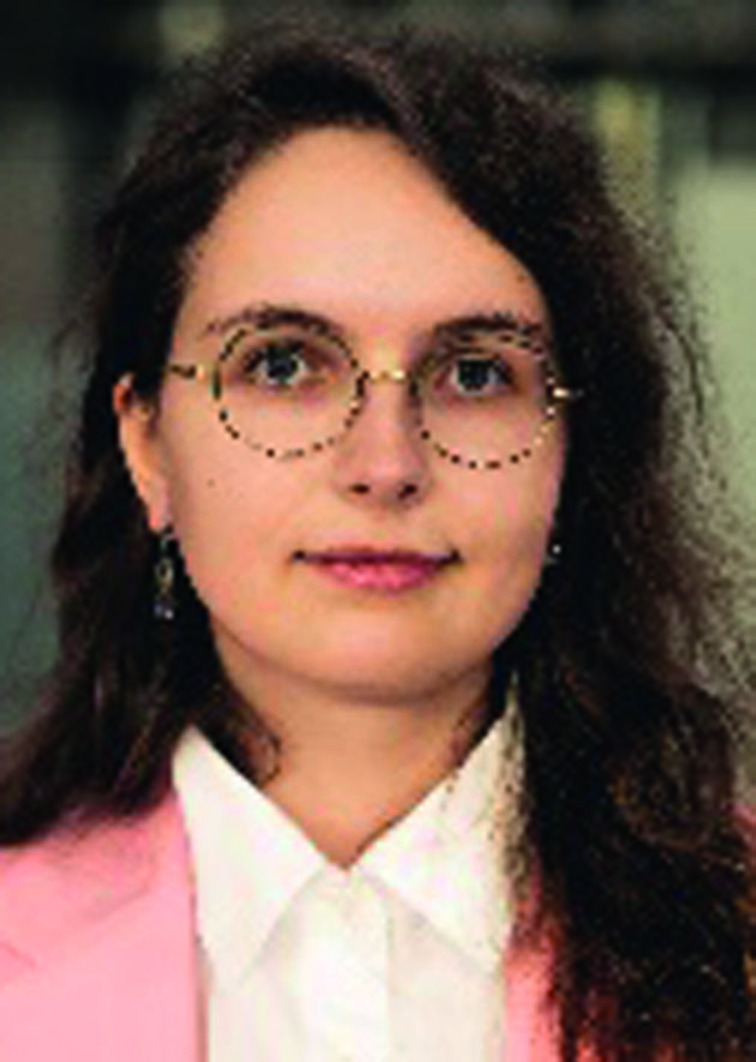

## Biographical Information

*Dr. Valery Radchenko is a tenured Research Scientist at TRIUMF and an adjunct professor at the University of British Columbia, Chemistry Department with a main research focus on the production and application of therapeutic radionuclides for radiopharmaceutical therapy. Radiochemist by training, he graduated from Saint‐Petersburg State Technical University in collaboration with the Joint Institute for Nuclear Research (JINR) in Dubna, received his PhD from Johannes‐Gutenberg University Mainz, Germany in 2013, followed by a postdoctoral position at Los Alamos National Laboratory, NM, USA*.

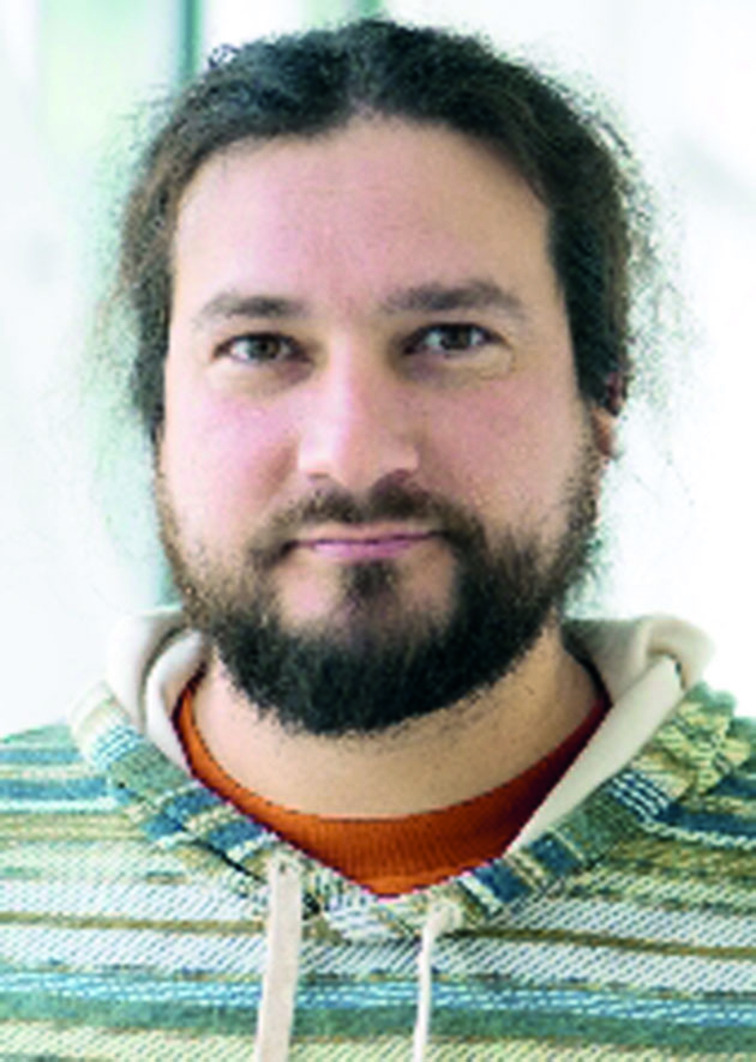

## Biographical Information

*Dr. Caterina Ramogida is an Assistant Professor in the Chemistry Department of Simon Fraser University (SFU) and holds a joint appointment with the Life Sciences Division at TRIUMF – Canada's particle accelerator center. She leads an interdisciplinary research program in Nuclear Medicinal Inorganic Chemistry, with a special interest in developing theranostic radiopharmaceuticals using exotic radiometals. She obtained her PhD in Medicinal Inorganic Chemistry from the University of British Columbia (2015), then joined the Life Sciences Division of TRIUMF as a postdoctoral research fellow in radiochemistry. In 2018, she joined SFU Chemistry and continues to work closely with TRIUMF*.

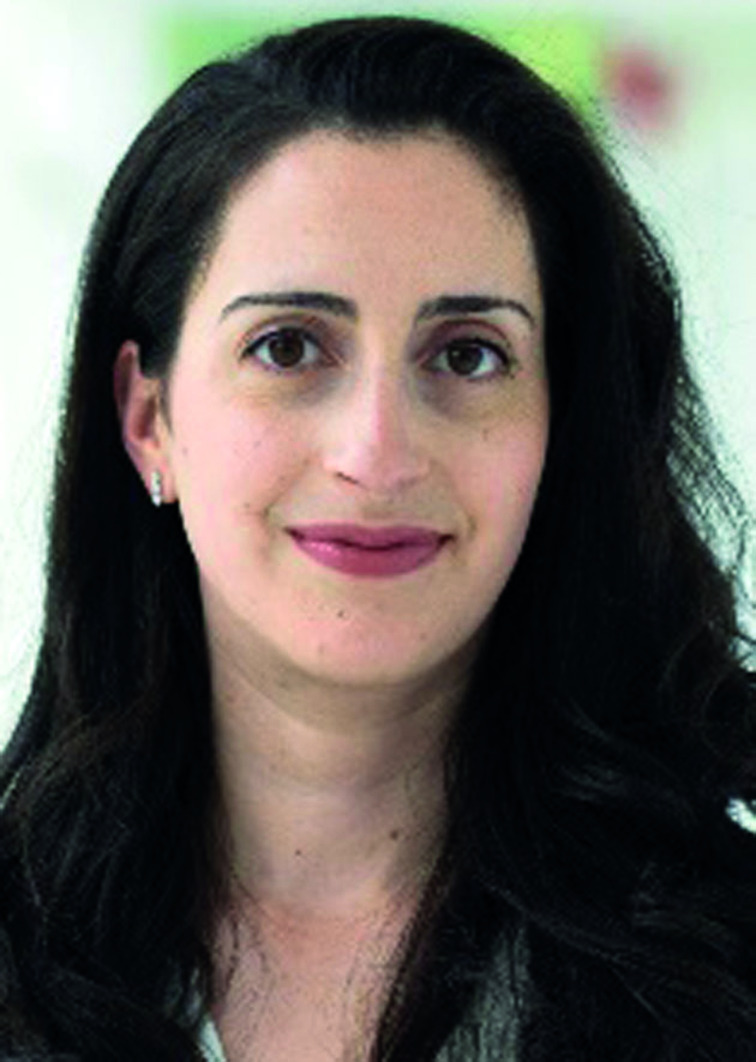
